# Therapeutic potential of pluripotent stem cell-derived dopaminergic progenitors in Parkinson’s disease: a systematic review protocol

**DOI:** 10.1186/s13643-021-01736-z

**Published:** 2021-06-25

**Authors:** Aliasghar Karimi, Mitra Elmi, Zahra Shiri, Hossein Baharvand

**Affiliations:** 1 Noncommunicable Diseases Research Center, Fasa University of Medical Sciences, Fasa, Iran; 2 Perron Institute for Neurological and Translational Science, QEII Medical Center, University of Western Australia, Perth, Australia; 3 Department of Stem Cells and Developmental Biology, Cell Science Research Center, Royan Institute for Stem Cell Biology and Technology, ACECR, Tehran, Iran; 4 Department of Developmental Biology, University of Science and Culture, Tehran, Iran; 5 Department of Brain and Cognitive Sciences, Cell Science Research Center, Royan Institute for Stem Cell Biology and Technology, ACECR, Tehran, Iran; 6 Royan Institute, Banihashem Sq., Banihashem St., Resalat Highway, 16635-148, Tehran, 1665659911 Iran

**Keywords:** Stem cell, Parkinson’s disease, Systematic review, Dopaminergic progenitors

## Abstract

**Background:**

Parkinson’s disease (PD) is the second most common age-dependent neurodegenerative disease that causes motor and cognitive disabilities. This disease is associated with a loss of dopamine content within the putamen, which stems from the degeneration of dopaminergic (DA) neurons in the substantia nigra pars compacta (SNc). Several approved drugs are available that can effectively treat symptoms of PD. However, long-term medical management is often complicated and does not delay or halt disease progression. Alternatively, cell replacement strategies can address these shortcomings and provide dopamine where it is needed. Although using human pluripotent stem cells (hPSCs) for treatment of PD is a promising alternative, no consensus in the literature pertains to efficacy concerns of hPSC-based therapy for PD. This systematic review aims to investigate the efficacy of primate PSC-derived DA progenitor transplantation to treat PD in preclinical studies.

**Methods:**

This is a systematic review of preclinical studies in animal models of PD. We intend to use the following databases as article sources: MEDLINE (via PubMed), Web of Science, and SCOPUS without any restrictions on language or publication status for all related articles published until the end of April 2021. Two independent reviewers will select the titles and abstracts, extract data from qualifying studies, and assess the risk of bias using the SYstematic Review Centre for Laboratory animal Experimentation (SYRCLE) risk of bias tool and the Collaborative Approach to Meta-Analysis and Review of Animal Data from Experimental Studies (CAMARADES) checklist. Apomorphine-induced rotation test (APO-IR) and amphetamine-induced rotation test (AMP-IR) are defined as the primary outcomes. The standardized mean difference (SMD) by Hedges’ g method (r) and odds ratio (OR) and related 95% confidence interval (CI) will be calculated to determine the size effect of the treatment. The heterogeneity between studies will be calculated by “I^2^ inconsistency of values and Cochran’s Q statistical test,” where I^2^ > 50% and/or *p* < 0.10 suggests high heterogeneity. Meta-analyses of random effects will be run when appropriate.

**Discussion:**

This study will present an overview of preclinical research on PSCs and their therapeutic effects in PD animal models. This systematic review will point out the strengths and limitations of studies in the current literature while encouraging the funding of new studies by public health managers and governmental bodies.

**Supplementary Information:**

The online version contains supplementary material available at 10.1186/s13643-021-01736-z.

## Background

Parkinson’s disease (PD) impacts 1% of the population above 60 years old; this presents an enormous economic and societal burden due to the global increase in aging. PD is a chronic neurodegenerative disease clinically diagnosed by tremor, rigidity, bradykinesia, cognitive disabilities, and other signs and symptoms that currently have no cure. Our understanding of the pathogenesis of PD suggests that inflammation, oxidative stress, excitotoxicity, mitochondrial dysfunction, and degeneration of dopaminergic (DA) neurons in the substantia nigra pars compacta (SNc) are to blame. The hallmark of the disease is the accumulation of Lewy bodies, which are inclusions of cytoplasmic proteins, mostly comprised of misfolded α-synuclein [1].

Treating PD is a challenge for clinicians as it is individualized and tailored for each patient. Surgical and pharmaceutical interventions are common, although they only temporarily mitigate the symptoms [2–4]. On the other hand, physical therapy without any intervention and medication can control PD in certain patients with minimal functional impairment [5]. Furthermore, studies suggest that exercise can help control some of the motor symptom of PD [6]. However, since the elderly make up most of the PD population, disease symptoms and other common disabilities prevent them from performing effective exercises.

Pharmaceutical interventions mostly consist of DA medications (e.g., Levodopa). However, long-term use of these medications causes significant adverse effects that include exacerbations of dyskinesia and drug resistance [7]. Deep brain stimulation (DBS) is the most common surgery used to treat PD for two decades. This method involves stimulation via implanted electrodes in the subthalamic nucleus and globus pallidus [8]. Although DBS can effectively manage certain PD symptoms, it is an expensive treatment that requires expensive device and medical care [9]. This device also limits patients as it raises concerns about battery life and LED migration. Furthermore, infections can arise from the implanted device which is cause for concern [10–12]. Aside from these concerns, DBS only controls the symptoms of PD for some time and does not address the progressive cell loss that occurs in the brain of these patients. On the other hand, cell therapy is less expensive and more effective as it replaces the lost cells and drastically halts the progression of the disease.

Immunotherapy is another approach where antibodies against α-synuclein are administered. However, immunotherapy raises the concern that reducing α-synuclein levels can halt normal protein function that leads to neurotoxicity [4]. Gene therapy has been considered in the treatment of PD. Specifically, trials have been conducted with AAV2-GAD gene therapy for advanced PD, but this approach seems to only be useful in genetic forms of PD [13–15].

The revolution in stem cell biology in the early 1980s opened up new avenues for many researchers and clinicians. Different types of pluripotent/multipotent cells are potentially used in preclinical and clinical studies. Pluripotent stem cells (PSCs), including embryonic stem cells (ESC), obtained from the inner cell mass of blastocysts and induced pluripotent stem cells (iPSC) derived by reprogramming somatic cells can differentiate to different cell types of interest, including DA neurons. These DA neurons have been used extensively in animal models of PD that were established using neurotoxins or pesticides. Such parkinsonism has been successfully treated with fetal midbrain grafts and ESC-derived DA cells [16–19].

As mentioned, the most recent developing treatment for PD is cell replacement therapy with a prospective long-term relief of disease symptoms. Many preclinical studies have investigated the therapeutic effects of PSC-DA neurons on PD animal models. The most widely used and well-established PD animal models are created by the administration of 6-hydroxydopamine (6-OHDA) or 1-methyl-4-phenyl-1,2,3,6-tetrahydropyridine (MPTP). Transplantation of human neural progenitor cells (NPCs) extracted from the midbrain of 14-week-old fetuses restored motor dysfunction in rats injected with 6-OHDA [3, 4, 16–19]. Other cell sources that proceeded to clinical trials in humans had poor clinical outcomes. These include medullary tissue, retinal pigmented epithelium cells, carotid body cells, and mesenchymal stem cells [4].

In contrast, promising clinical trials have been conducted that involved the implantation of midbrain DA progenitors from the human fetal brain to individuals with PD. However, the use of fetal tissue poses several problems—low availability, high variability, and ethical concerns that differ (are different) between countries. Therefore, Researchers and clinicians have searched for alternate cell sources. iPSCs or ESC-derived DA progenitor cells appear to be the most suitable alternative to generate ventral mesencephalic DA progenitors for transplantation in PD [20].

Existing reviews of stem cell therapy in PD focused on mesenchymal stem cells, which have the disadvantages of modest clinical outcomes and insufficient sources of embryonic tissues [21–23]. Therefore, in this systematic review, we intend to comprehensively examine the therapeutic effects of human and non-human primate PSC-derived DA progenitors in rats, mice, and monkeys with PD. Treatment outcomes will be evaluated by obtaining data from the various behavioral tests performed. We aim to examine the efficacy of primate (human and non-human) PSC-derived DA progenitor transplantation for treating PD in preclinical studies as an outlook for launching clinical trials.

## Methods

The protocol of this systematic review will adhere to the desired anecdote matters for systematic reviews and meta-analyses for protocols Preferred Reporting Items for Systematic Review and Meta-analysis Protocols (PRISMA-P), recommendations for reporting of systematic reviews and meta-analyses of animal experiments [24–26]. The PRISMA-P checklist has seventeen main items to ease systematic reviews. This checklist guides authors to organize their review in terms of administrative information, introduction, and methods [24]. The PRISMA-P details and updates are available at http://www.prisma-statement.org/Extensions/Protocols.aspx.

This protocol is registered at the International Prospective Register of Systematic Reviews (PROSPERO—CRD42020168304) at https://www.crd.york.ac.uk/PROSPERO/display_record.php?RecordID=168304.

### Eligibility criteria

#### Types of studies

All animal intervention studies that included a control group will be enrolled in this systematic review, which evaluate primate PSC-derived DA progenitor transplantation in preclinical animal models of PD.

#### Types of animal models

All studies that used animal models of experimental PD (6-OHDA or MPTP) in which the animals developed characteristic motor deficits will be considered in this systematic review.

#### Types of comparators

The comparison group includes animal models of PD that did not receive cells.

#### Types of intervention

The intervention group includes animals that received human or non-human primate PSC-derived DA progenitors and have been investigated for the treatment of PD.

Only studies that used iPSCs and ESCs of a primate origin to derive DA progenitors, neural stem cells (NSCs), or NPCs following the establishment of the PD model will be included in the study.

### Exclusion criteria

Non-intervention studies such as case reports, congress abstracts, letters to the editor, human studies, and studies that include in vitro experiments only will be excluded. Studies that do not evaluate behavior as an outcome will be excluded, in addition to studies that included PSCs from a non-primate source. Also, non-English papers will be excluded.

### Types of outcome measures

The primary outcomes are defined as the rescued motor deficits as measured by drug-induced rotation tests in rodents and PD scores in primate studies. Specifically, apomorphine-induced rotation test (APO-IR) and Amphetamine-induced rotation test (AMP-IR) measure motor behavior in rodents by counting the number of net turns towards the lesion (amphetamine) or away from the lesion (apomorphine) per min in a 60–90-min test following subcutaneous injection of the respective  stimulant drug. A score of greater than 5 indicates the rodent is hemi-parkinsonian.

In primate models of the disease, a Parkinson score is assigned to each animal based on a list of physical assessments. The scale depends on the scoring chart being used and there usually is not a threshold score to denote a parkinsonian animal. In these assessments, a decrease in parkinsonian score indicates a rescue of motor deficits.

The secondary outcomes are described below:

The cylinder test assesses the symmetry in spontaneous use of forelimbs in rodents. In this test, the use of the contralateral forelimb is counted in a 10-min test and a percentage of contralateral limb use is used to indicate a hemi-parkinsonian rodent. A score of 50% is indicative of no motor symptoms. Neurological scores are a subjective assessment based on a series of observations where a score of 0 is normal and a score of 1 is abnormal. Spontaneous movement, spontaneous rotation, and time in movement are subjective assessments of parkinsonian motor symptoms where abnormal behavior can be noted. The rotarod test measures a rodent’s ability to balance on a rotating rod as it moves across.

Histological data measure the fate of transplanted cells in the graft recipient by locating the cells in the brain and using various markers to assess cell fate. Finally, neuroimaging data obtained before and after cell injections also show cell fate and can measure the amount of dopamine release in the striatum.

### Searching methods for identification of studies

#### Electronic searches

Electronic databases used by MEDLINE via PubMed, Web of Science, and SCOPUS from their foundation until the end of April 2021 without any search filters and restrictions of language, date of publication, or publication status are utilized.

#### Search strategy

The main terms are as follows: “pluripotent stem cell” OR “dopaminergic progenitor” OR “DA progenitor” OR “neural stem cell” OR NSC OR “neural progenitor” OR NPC OR “embryonic stem cell” OR ESC OR “induced pluripotent stem cell” OR iPSC OR “pluripotent stem cell” OR PSC) AND (Parkinson’s). Supplement 1 lists the details of search strategies to be used in the electronic databases.

### Data collection and analysis

#### Selection of studies

All studies are imported into Covidence, which is a not-for-profit service established in 2013, and run by a team in Melbourne, Australia [27]. At first, duplicate studies will be removed by Covidence. Then, two reviewers (AAK and ZS) will independently screen titles and abstracts. Any conflicts between reviewers will be resolved by agreement or by a third reviewer (HB). After screening the titles and abstracts, the same reviewers will independently evaluate the full text of the studies by using a standardized form that contains the inclusion and exclusion criteria.

#### Data extraction

Two reviewers (ZS and ME) will independently extract all available sources in the text and graphs of each article. If only graphical data are available, values for mean and standard deviation or standard error will be obtained. Since the graphs are often simple bar charts, each reviewer independently reads the means and SDs at higher magnification by ruler tool from Adobe® Acrobat Reader software. The calibration exercises will be conducted to ensure consistency between reviewers before starting data extraction.

The data will be extracted using standardized extraction forms: (1) study characteristics (author, year of publication); (2) features of the included animals and animal models (animal species, PD model, age, gender, farming situations, numbers of animals in intervention, and comparison group); (3) interventions (time and description of preparation); and (4) outcomes of interest.

#### Risk of Bias assessment

Two reviewers (ZS and ME) will independently use the SYstematic Review Centre for Laboratory animal Experimentation (SYRCLE) risk of bias tool to evaluate the quality of the studies and risk of any bias. The SYRCLE risk of bias tool includes ten defined criterion assessment domains related to biases of selection, performance, identification, attrition, and reporting. For each included study, all domains will be scored as low, high, or unclear risk of bias [28].

The Collaborative Approach to Meta-Analysis and Review of Animal Data from Experimental Studies (CAMARADES) checklist will be used to evaluate the selected studies. This checklist has 11 items: publication in a peer-reviewed journal, recording of temperature control, randomized treatment allotment, blinded evaluation of results, reporting of blinding of the operator, suitable animal models, reporting of a sample size calculation, agreement with animal wellbeing principles, statement of potential conflict of interest, and a comprehensive follow-up.

All studies are evaluated by this checklist. Each item receives a score of 0 or 1 (No or Yes) such that a study with more items included has a higher score. Thus, studies can be compared and a study with a higher overall score has a lower risk of bias [29, 30].

#### Assessment of the treatment efficacy

Primary endpoints are determined before cell transplantation and the secondary endpoints relate to the end of the study. The effect size will be measured by Hedges’ g [31] method (r) so that the mean difference of outcome between primary and secondary endpoint of intervention group compared with the control group.

Standardized mean difference (SMD) will be used for continuous data and the dichotomous data will be evaluated by odds ratio (OR) and related 95% confidence interval (CI).

#### Data synthesis

In the first step of data synthesis, all characteristics of studies are summarized in a table such that similar studies based on animal models are defined as the sub groups. We will then pool the continuous outcomes using the ratio of weighted means method to adjust different units and compare effect sizes between intervention and control groups. The weight equals the inverse of the modified variance of size effect. This method is suitable for small sample sizes in animal studies and is similar to a risk ratio so that clinical interpretation by this method is simple.

Also, we will use the OR and 95% CI to describe and pool the dichotomous data. If more than three papers are available, we will perform the meta-analysis with inverse variance random effects modeling [32].

#### Assessment of heterogeneity

The heterogeneity between studies will be estimated by calculating “I^2^ inconsistency values and Cochran’s Q statistical test,” where I^2^ > 50% and/or *p* < 0.10 recommends high heterogeneity. Heterogeneity will be defined according to the I^2^ range: 0–40% (minor heterogeneity), 40–60% (moderate heterogeneity), 60–90% (substantial heterogeneity), and >90% (significant heterogeneity) [33, 34].

The analyses will be performed using Review Manager 5.3 [35]. In cases where the Review Manager statistical software is not sufficient, data analyses will be performed by STATA® statistical software, version 14.2 (Stata Corp., College Station, TX, USA) [36].

#### Assessment of publication bias

Egger’s regression asymmetry test via graphical funnel plot will be used. All studies contribute to a pooled analysis to obtain the linear regression of the intervention size effect, which will be presented in the studies. The highly asymmetric graphical funnel plot shows a significant publication bias [37, 38].

## Discussion

This study will provide clinicians and researchers with evidence of preclinical research and relevant evidence that pertains to the therapeutic potential of PSC-derived DA progenitor transplantation for PD. This study intends to show the strengths and limitations of the previous studies to suggest future outlooks in this field. Although numerous experimental studies about the effects of PSC-based therapy for PD have been published, there is no consensus in the literature. Therefore, a systematic analysis of existing experimental studies is essential as a perspective for launching clinical trials.

## Supplementary Information

**Additional file 1.** Search Strategies.

**Additional file 2.** PRISMA-P checklist.

## Data Availability

Not applicable.

## Abbreviations

PD: Parkinson’s disease
DA: Dopaminergic
hPSCs: Human pluripotent stem cells
PSCs: Pluripotent stem cells
ESC: Embryonic stem cells
iPSC: Induced pluripotent stem cells
NSCs: Neural stem cells
95% CI: 95% confidence intervals
CAMARADES: Collaborative Approach to Meta-Analysis and Review of Animal Data from Experimental Studies
MeSH: Medical Subject Headings
OR: Odds ratio
PRISMA-P: Preferred Reporting Items for Systematic reviews and Meta-Analyses for Protocols
PROSPERO: International Prospective Register of Systematic Reviews
RR: Risk ratio
SMD: Standardized mean difference
SYRCLE: SYstematic Review Center for Laboratory animal Experimentation
