# Supplementary material for: Therapeutic potential of pluripotent stem cell-derived dopaminergic progenitors in Parkinson’s disease: a systematic review protocol
Source: Syst Rev. 2021 Jun 25;10:188. doi: 10.1186/s13643-021-01736-z (PMC8235644; doi:10.1186/s13643-021-01736-z)
Supplement: Supplementary file 1 — Additional file 1. Search Strategies. [file 13643_2021_1736_MOESM1_ESM.docx]

**Supplement 1.** Search Strategies

**Pubmed details:**

("pluripotent stem cell"[All Fields] OR (("dopamine"[MeSH Terms] OR "dopamine"[All Fields] OR "dopaminergic"[All Fields]) AND progenitor[All Fields]) OR (DA[All Fields] AND progenitor[All Fields]) OR "neural stem cell"[All Fields] OR NSC[All Fields] OR "neural progenitor"[All Fields] OR ("Nat Prod Commun"[Journal] OR "npc"[All Fields]) OR "embryonic stem cell"[All Fields] OR ESC[All Fields] OR "induced pluripotent stem cell"[All Fields] OR ("induced pluripotent stem cells"[MeSH Terms] OR ("induced"[All Fields] AND "pluripotent"[All Fields] AND "stem"[All Fields] AND "cells"[All Fields]) OR "induced pluripotent stem cells"[All Fields] OR "ipsc"[All Fields]) OR "pluripotent stem cell"[All Fields] OR PSC[All Fields]) AND Parkinson[All Fields]

**Scopus details:**

( TITLE-ABS-KEY ( ( "pluripotent stem cell" OR "dopaminergic progenitor" OR "DA progenitor" OR "neural stem cell" OR nsc OR "neural progenitor" OR npc OR "embryonic stem cell" OR esc OR "induced pluripotent stem cell" OR ipsc OR "pluripotent stem cell" OR psc ) ) AND TITLE-ABS-KEY ( parkinson ) )

**Web of Science details:**

ALL= (("pluripotent stem cell" OR "dopaminergic progenitor" OR "DA progenitor" OR "neural stem cell" OR NSC OR "neural progenitor" OR NPC OR "embryonic stem cell" OR ESC OR "induced pluripotent stem cell" OR ipsc OR "pluripotent stem cell" OR PSC) AND Parkinson)

Timespan: All years. Indexes: SCI-EXPANDED, SSCI, CPCI-S, CPCI-SSH, ESCI.
